# Effectiveness of the Tier 1 Program of Project P.A.T.H.S.: Findings Based on the First 2 Years of Program Implementation

**DOI:** 10.1100/tsw.2009.66

**Published:** 2009-07-01

**Authors:** Daniel T. L. Shek

**Affiliations:** ^1^ Department of Applied Social Sciences, The Hong Kong Polytechnic University, Hong Kong, China; ^2^ Department of Sociology, East China Normal University, Shanghai, China; ^3^ Kiang Wu Nursing College of Macau, Macau, China

**Keywords:** adolescents, positive youth development, randomized group trial, P.A.T.H.S., objective outcome evaluation

## Abstract

The Tier 1 Program of the Project P.A.T.H.S. (Positive Adolescent Training through Holistic Social Programmes) is a curricular-based program that attempts to promote positive youth development in Hong Kong. In the second year of the Full Implementation Phase, 20 experimental schools (N = 2,784 students) and 23 control schools (N = 3,401 students) participated in a randomized group trial. Analyses of covariance and linear mixed models, controlling for differences between the two groups in terms of Wave 1 pretest scores, personal variables, and random effect of schools, showed that participants in the experimental schools had significantly higher positive youth development levels than did participants in the control schools at post-test, based on different indicators derived from the Chinese Positive Youth Development Scale. The students in the experimental schools also displayed a lower level of delinquency, but better school adjustment than did students in the control schools. Differences between experimental and control participants were also found when students who joined the Tier 1 Program and perceived the program to be beneficial were employed as participants of the experimental schools.

### INTRODUCTION

In different parts of the world, policy makers and professionals in the education and welfare fields are concerned about the development of young people. In particular, helping professionals attempt to develop programs that are effective in promoting the positive development of adolescents. A survey of the literature shows that there are many positive youth development and adolescent prevention programs in the field. Obviously, one basic question is whether or not the existing programs are effective in promoting positive youth development. Catalano and associates[1] reviewed the effectiveness of 77 positive youth development programs. Results showed that only 25 programs were successful and different positive youth development constructs were incorporated into the successful programs. These constructs include:

promotion of bonding, cultivation of resilience, promotion of social competence, promotion of emotional competence, promotion of cognitive competence, promotion of behavioral competence, promotion of moral competence, cultivation of self-determination, promotion of spirituality, development of self-efficacy, development of a clear and positive identity, promotion of beliefs in the future, provision of recognition for positive behavior, provision of opportunities for prosocial involvement, and fostering prosocial norms.

With particular reference to Hong Kong, there are very few multiyear positive youth development programs for early adolescents. Although some schools in Hong Kong offer courses on personal development in the names of moral education, civic education, or life education, validated and effective programs are almost nonexistent. In view of this gap in the field and to promote holistic development in adolescents in Hong Kong, The Hong Kong Jockey Club Charities Trust invited academics of five local universities to form a research team, with the author being the Principal Investigator, to develop a multiyear universal positive youth development program in the territory (Project P.A.T.H.S. [Positive Adolescent Training through Holistic Social Programmes]). There are two tiers of programs in this project. The Tier 1 Program is a universal positive youth development program designed for Secondary 1 to 3 students (i.e., universal prevention). There are 10 and 20 h of training for the core program and full program in each school year for each grade, respectively. The Tier 2 Program is specifically designed for students who display greater psychosocial needs at each grade (i.e., selective prevention). To help adolescents to develop in a holistic manner, the 15 adolescent developmental constructs listed above are covered in the Project P.A.T.H.S., particularly in the Tier 1 Program. The design of the program can be seen in the publications of the project[2,3].

To provide a comprehensive picture regarding the effectiveness of the project, evaluation strategies based on the principle of triangulation have been used to evaluate the effectiveness of the project. These include objective outcome evaluation, subjective outcome evaluation based on the perspectives of the program participants and program implementers, qualitative evaluation based on focus groups, student diaries and in-depth interviews, process evaluation, and interim evaluation. To date, research findings generally suggest that program implementers and participants perceived the program as beneficial to the development of the program participants; program participants displayed positive changes in development after joining the program[4,5,6].

Objective outcome evaluation has been regarded as the "gold standard" for evaluation of social intervention programs. Based on this perspective, research findings showed that there were positive changes in the program participants after joining the program. Based on a one-group pre/post-test design, Shek[7] concluded that the participants showed positive changes in development in several domains of positive youth development after joining the program. Based on a randomized group trial using the data collected at Secondary 1 (i.e., Waves 1 and 2 data), Shek and associates[8] found that compared with the control group participants, experimental group participants showed greater positive changes in psychosocial competencies and global positive youth development. As the findings reported in the previous studies were limited to Secondary 1 students only, there is a need to examine the effectiveness of the P.A.T.H.S. Program over a longer period of time. As such, differences between participants in the experimental and control groups are examined with reference to both Secondary 1 (Waves 1 and 2) and Secondary 2 data (Waves 3 and 4) in this paper. Besides, as previous findings showed that roughly one-fifth of the participants did not find the program to be helpful[7], it would be insightful to examine the differences between those experimental participants who found the program to be beneficial and the control participants. The general hypothesis is that participants in the experimental group (particularly those perceiving the program to be effective) should perform better than the control participants.

## METHODS

### Participants and Procedures

Shek[7] described the procedures and criteria for recruiting the initial 24 experimental schools (one school dropped out after pretest) and 24 control schools in Year 1, during which the Waves 1 and 2 data were collected from Secondary 1 students. In Year 2, Waves 3 and 4 data were collected from the same cohort promoting to Secondary 2, with 20 experimental schools and 23 control schools. The design of the study in Years 1 and 2 is presented in Table 1. The number of students who joined the experimental and control groups in Years 1 and 2 and the number of completed questionnaires collected can be seen in Table 2.

**TABLE 1**
**Experimental and Control Schools at Year 1 (Secondary 1) and Year 2 (Secondary 2)**

| Pair | Year 1 | | Year 2 | | Characteristics |
|---|---|---|---|---|---|
| | Experimental Group | Control Group | Experimental Group | Control Group | |
| Pair 1 | 1A | 1B | 1A | 1B | Nineteen pairs of schools with identical school banding. In each pair, one school was randomly assigned to the experimental group and one school was randomly assigned to the control group. |
| Pair 2 | 2A | 2B | 2A | 2B | |
| Pair 3 | 3A | 3B | 3A | 3B | |
| Pair 4 | 4A | 4B | 4A | 4B | |
| Pair 5 | 5A | 5B | 5A | 5B | |
| Pair 6 | 6A | 6B | 6A | 6B | |
| Pair 7 | 7A | 7B | 7A | 7B | |
| Pair 8 | 8A | 8B | 8A | 8B | |
| Pair 9 | 9A | 9B | 9A | 9B | |
| Pair 10 | 10A | 10B | 10A | 10B | |
| Pair 11 | 11A | 11B | 11A | 11B | |
| Pair 12 | 12A | 12B | 12A | 12B | |
| Pair 13 | 13A | 13B | 13A | 13B | |
| Pair 14 | 14A | 14B | 14A | 14B | |
| Pair 15 | 15A | 15B | 15A | 15B | |
| Pair 16 | 16A | 16B | 16A | 16B | |
| Pair 17 | 17A | 17B | 17A | 17B | |
| Pair 18 | 18A | 18B | 18A | 18B | |
| Pair 19 | 19A | 19B | 19A | 19B | |
| Pair 20 | 20A | 20B | 20A | 20B | Four pairs of schools with similar banding and randomly assigned. |
| Pair 21 | 21A | 21B | 21A | 21B | |
| Pair 22 | 22A | 22B | 22A | 22B | |
| Pair 23 | 23A | 23B | 23A | 23B | |
| Pair 24 | 24A | N/A | 24A | N/A | One pair of schools with similar banding, but not randomly assigned. |
| | N/A | 24B | N/A | 24B | |

*Note*: Initially, at Year 1, there were 24 experimental schools (one school completed pretest only) and 24 control schools. Schools that withdrew at Year 2 are marked with shading.

**TABLE 2**
**Number of Participants and Completed Questionnaires Collected at Year 1 (Waves 1 and 2) and Year 2 (Waves 3 and 4)**

| Cases | Year 1 | | | Year 2 | | |
|---|---|---|---|---|---|---|
| | Experimental | Control | Total | Experimental | Control | Total |
| Pretest questionnaire collected | 4,121 | 3,854 | 7,975 | 3,290 | 3,861 | 7,151 |
| Pretest questionnaire available for matching | 4,050 | 3,795 | 7,845 | 3,276 | 3,845 | 7,121 |
| Post-test questionnaire collected | 3,914 | 3,770 | 7,684 | 3,047 | 3,764 | 6,811 |
| Post-test questionnaire available for matching | 3,880 | 3,728 | 7,608 | 3,047 | 3,763 | 6,810 |
| Successfully matched | 3,312 (49.6%) | 3,363 (50.4%) | 6,675 (100%) | 2,784 (45.0%) | 3,401 (55.0%) | 6,185 (100%) |

*Note*: The number (percentage) of the successfully matched cases across Waves 1–4 is 5,054 (100%): experimental group, 2,236 (44.2%); control group, 2,818 (55.8%).

At pre- and post-test, the purpose of the study was mentioned, and confidentiality of the data collected was repeatedly emphasized to all students in attendance on the day of testing. Parental and student consent had been obtained prior to data collection. All participants responded to all scales in the questionnaire in a self-administration format. Adequate time was provided for the participants to complete the questionnaire. A trained research assistant was present throughout the administration process.

### Instruments

Consistent with the procedures used in Year 1, the participants were invited to respond to a questionnaire that contained different measures of youth development at pretest (i.e., before the program began) and post-test (i.e., after the program ended). The following measures were used.

#### *Chinese Positive Youth Development Scale (CPYDS)*

Based on the analyses conducted in Year 1, the item composition of the 15 subscales of the CPYDS is listed as follows:

1. Bonding Subscale (six items): $\alpha = 0.86$ and 0.88 at pre- and post-test.
2. Resilience Subscale (six items): $\alpha = 0.88$ and 0.88 at pre- and post-test.
3. Social Competence Subscale (seven items): $\alpha = 0.87$ and 0.87 at pre- and post-test.
4. Emotional Competence Subscale (six items): $\alpha = 0.86$ and 0.86 at pre- and post-test.
5. Cognitive Competence Subscale (six items): $\alpha = 0.87$ and 0.88 at pre- and post-test.
6. Behavioral Competence Subscale (modified five items): $\alpha = 0.82$ and 0.82 at pre- and post-test.
7. Moral Competence Subscale (six items): $\alpha = 0.81$ and 0.80 at pre- and post-test.
8. Self-Determination Subscale (five items): $\alpha = 0.82$ and 0.81 at pre- and post-test.
9. Self-Efficiency Subscale (modified two items): $\alpha = 0.58$ and 0.59 at pre- and post-test.
10. Beliefs in the Future Subscale (modified three items): $\alpha = 0.84$ and 0.84 at pre- and post-test.
11. Clear and Positive Identity Subscale (seven items): $\alpha = 0.87$ and 0.86 at pre- and post-test.

12. Spirituality Subscale (seven items): α = 0.91 and 0.91 at pre- and post-test.
13. Prosocial Involvement Subscale (five items): α = 0.86 and 0.85 at pre- and post-test.
14. Prosocial Norms Subscale (five items): α = 0.81 and 0.81 at pre- and post-test.
15. Recognition for Positive Behavior Subscale (four items): α = 0.83 and 0.83 at pre- and post-test.

Several composite indices based on the above measures were also formed to give a picture of the intervention. First and foremost, according to Shek[7], the mean of the total mean score based on 12 subscales (excluding behavioral competence, self-determination, prosocial norms) could be used as an overall measure of positive youth development (CPYDS-12: α = 0.94 and 0.93 at pre- and post-test). Next, as it can be argued that constructs including spirituality, prosocial norms, prosocial involvement, bonding, and recognition for positive behavior are different from the rest of the scales, a summation of 10 subscales (CPYDS-10: α = 0.94 and 0.94 at pre- and post-test) assessing psychosocial competence and strengths was used (i.e., resilience, social competence, emotional competence, cognitive competence, behavioral competence, moral competence, self-determination, self-efficiency, beliefs in the future, and clear and positive identity). Third, based on conceptual analyses of the items, one key item was derived for each domain, which resulted in a 15-item key measure (KEY15: α = 0.90 and 0.90 at pre- and post-test). Fourth, based on item analysis, a 36-item measure was derived (KEY36: α = 0.96 and 0.96 at pre- and post-test). Finally, based on factor analysis (the details of which can be obtained from the author), 18 items based on the first two factors involving items of social, emotional, and cognitive competencies were used (SEC: α = 0.94 and 0.94 at pre- and post-test).

#### *Delinquency Scale (DE)*

Twelve items were used to assess the frequency of delinquent behavior in the past year, including stealing, cheating, playing truant, running away from home, damaging others' properties, assault, sexual relationship, gang fighting, speaking foul language, staying away from home without parental consent, "strong arming" others, and breaking into others' properties. The present findings show that the measure was internally consistent at pretest (α = 0.79) and post-test (α = 0.82) in Year 2.

#### *School Adjustment Measures (SA)*

Three items were used to assess the school adjustment of the participants. The first item assessed a respondent's perception of his or her academic performance when compared with schoolmates in the same grade. The respondents were asked to rate "Best", "Better than usual", "Ordinary", "Worse than usual", or "Worst" in this item. The second item assessed the respondent's satisfaction with his or her academic performance. The respondents were asked to rate "Very satisfied", "Satisfied", "Average", "Dissatisfied", or "Very Dissatisfied" in this item. The final item assessed the respondent's perception of his or her conduct. The respondents were asked to rate "Very good", "Good", "Average", "Poor", or "Very Poor" in this item. Previous research findings showed that these three items and the related scale were temporally stable and valid[9]. The present findings showed that the measure was internally consistent at pretest (α = 0.72) and post-test (α = 0.73) in Year 2.

#### *Subjective Outcomes Scale (SOS)*

Twenty items were used to assess the participant's satisfaction with the course and instructor as well as their perceived benefits of the program at post-test (i.e., Wave 4 data). The response options included "Strongly disagree", "Moderately disagree", "Slightly disagree", "Slightly agree", "Moderately agree", and “Strongly agree”. Reliability analysis showed that this measure is reliable ($\alpha = 0.97$). Item 20 of this scale is “Overall speaking, the program was beneficial to my development”.

### Data Analytic Strategies

Allison et al.[10] pointed out that there were four basic strategies in analyzing change data associated with experimental designs. The first strategy was to examine the difference between the experimental and control groups at post-test only. As this strategy did not take into account all information, it was not a recommended approach. The second strategy was to conduct a two-way ANOVA (with group and time as the main effect) and examine the interaction effect between group and time. As this approach was often misinterpreted, it was also not recommended. The third strategy was to look at gain scores. However, as the correlation between pre- and post-test scores seldom equals to 1.0, there would be bias in this analysis. The final recommended strategy was to use analyses of covariance to compare post-test scores of the experimental and control groups after controlling pre-test scores. In this study, the final strategy based on analysis of covariance with Wave 4 outcomes as dependent variables controlling the Wave 1 baseline scores was used. Furthermore, as students in this study were recruited from schools, it could be argued that variations in the outcome measures across groups may also be due to variations in the school characteristics across groups. As such, there is a need to adjust for the random effect of schools when examining the effect of treatment on the outcome variables[11,12]. SPSS linear mixed models were used to conduct the related analyses[13].

## RESULTS

Using schools as the units of analysis, results showed that the 20 experimental schools and 23 control schools did not differ in the banding of the schools, districts, religion, gender of the students, and source of funding. For the personal characteristics of the participants, results showed that there were no statistically significant differences between the two groups in their background sociodemographic characteristics ($p > 0.05$ in all cases), except age. In short, except that the mean age of the control group was higher than that of the experimental group, the background characteristics of the experimental and control schools were highly comparable at Wave 1.

For the findings based on analyses of covariance controlling for pretest scores at Wave 1, results in Table 3 show that there were significant differences between the experimental and control group participants in terms of the five global indicators. For the linear mixed models, the hypothesized models were significantly better than the intercept models, with findings based on the hypothesized models as generally positive. The findings generally showed that the experimental group performed better than the control group in terms of the global positive youth development and school adjustment indicators (CPYDS-12, CPYDS-10, KEY15, KEY36, SEC, and SA), after controlling for pretest scores and age, as well as adjusting for the random effect of schools.

Further analyses based on experimental participants who found the program to be beneficial (responding in the direction of agreement to SOS item 20) vs. control participants similarly showed that the experimental participants generally performed better than control participants in terms of the global positive youth development indicators (Table 4). Furthermore, the experimental participants with the above characteristics showed a lower level of delinquency.

## DISCUSSION

The purpose of this paper is to report objective outcome evaluation findings regarding the effectiveness of a positive youth development program (Project P.A.T.H.S.) in Hong Kong. There are several unique features

**TABLE 3**
**Differences between the Experimental (Joining the Tier 1 Program Only) and Control Group based on the Different Indicators Derived from the Chinese Positive Youth Development Scale**

| Global Indicator | Estimated Marginal Mean (Control Group) | Estimated Marginal Mean (Experimental Group) | *F* Value |
|---|---|---|---|
| Findings Based on Analyses of Covariance | | | |
| CPYDS-12 | 4.431 | 4.475 | 7.43, $p < 0.01$ |
| CPYDS-10 | 4.422 | 4.466 | 7.45, $p < 0.01$ |
| KEY15 | 4.378 | 4.432 | 10.16, $p < 0.005$ |
| KEY36 | 155.281 | 157.515 | 11.16, $p < 0.005$ |
| SEC | 4.391 | 4.446 | 9.59, $p < 0.005$ |
| DE | 0.516 | 0.482 | 3.82, $p < 0.05$ (one tailed) |
| SA | 2.973 | 3.049 | 15.22, $p < 0.001$ |
| Linear Mixed Model Findings Adjusting for the Random Effect of Schools | | | |
| CPYDS-12 | 4.431 | 4.473 | 2.57, $p < 0.06$ (one tailed) |
| CPYDS-10 | 4.421 | 4.466 | 2.89, $p < 0.05$ (one tailed) |
| KEY15 | 4.377 | 4.430 | 3.77, $p < 0.05$ (one tailed) |
| KEY36 | 155.400 | 157.613 | 4.33, $p < 0.05$ |
| SEC | 4.390 | 4.448 | 4.18, $p < 0.05$ |
| DE | 0.504 | 0.494 | 0.08, ns |
| SA | 2.981 | 3.052 | 3.77, $p < 0.05$ (one tailed) |

*Note*: CPYDS-12: global indicator comprising bonding, resilience, social competence, emotional competence, cognitive competence, moral competence, self-efficacy, beliefs about the future, spirituality, clear and positive identity, prosocial involvement, and recognition for positive behavior. CPYDS-10: global indicator comprising resilience, social competence, emotional competence, cognitive competence, behavioral competence, moral competence, self-determination, self-efficacy, beliefs in the future, and clear and positive identity. KEY15: indicator based on 15 key items. KEY36: indicator based on 36 items. SEC: social-emotional competence indicator based on Factor 1 and Factor 2. DE: Delinquency Scale. SA: school adjustment behavior.

of the study. First, a large sample size was used so as to enhance the power of analyses. Second, the schools were randomly assigned to the experimental and control groups to minimize differences in the two groups. Third, a validated measure (Chinese Positive Youth Development Scale) with different global measures of positive youth development was used. Fourth, both Secondary 1 and 2 data (i.e., Wave 1 data as pretest and Wave 4 data as post-test, respectively) were used in the analyses. Fifth, both analyses of covariance and linear mixed models were used to analyze the data. Finally, this is the first known scientific study that adopted a randomized group trial design using data spanning over 2 years to evaluate a positive youth development program based on a curricular approach in different Chinese communities.

The findings generally showed that compared with participants in the control group, participants in the experimental schools performed better in different indicators of positive youth development. In particular, the findings revealed that experimental participants performed better than control participants in the areas of psychosocial competencies. For example, findings based on CPYDS-10 suggest that the experimental subjects displayed higher scores on the global indicator reflecting resilience, social competence, emotional competence, cognitive competence, behavioral competence, moral competence, self-determination, self-efficacy, beliefs in the future, and clear and positive identity. On the other hand, participants in the experimental schools displayed a lower level of delinquency, but better school adjustment than did participants in the control schools. Further analyses based on the experimental subjects who found the program to be beneficial to their development only (i.e., response to SOS item 20 in the positive direction)

**TABLE 4**
**Differences between the Experimental (Joining the Tier 1 Program Only and Perceiving the Program to be Beneficial to Their Own Development) and Control Group based on the Different Indicators Derived from the Chinese Positive Youth Development Scale**

| Global Indicator | Estimated Marginal Mean (Control Group) | Estimated Marginal Mean (Experimental Group) | *F* Value |
|---|---|---|---|
| Findings Based on Analyses of Covariance | | | |
| CPYDS-12 | 4.442 | 4.547 | 41.28, $p < 0.0001$ |
| CPYDS-10 | 4.431 | 4.527 | 34.81, $p < 0.0001$ |
| KEY15 | 4.386 | 4.501 | 44.67, $p < 0.0001$ |
| KEY36 | 155.60 | 159.701 | 36.48, $p < 0.0001$ |
| SEC | 4.397 | 4.510 | 39.18, $p < 0.0001$ |
| DE | 0.513 | 0.447 | 14.07, $p < 0.0001$ |
| SA | 2.979 | 3.090 | 31.03, $p < 0.0001$ |
| Linear Mixed Model Findings Adjusting for the Random Effect of Schools | | | |
| CPYDS-12 | 4.441 | 4.548 | 20.04, $p < 0.0001$ |
| CPYDS-10 | 4.430 | 4.528 | 17.88, $p < 0.0001$ |
| KEY15 | 4.385 | 4.501 | 21.72, $p < 0.0001$ |
| KEY36 | 155.704 | 1597.847 | 16.48, $p < 0.0001$ |
| SEC | 4.396 | 4.512 | 21.18, $p < 0.0001$ |
| DE | 0.501 | 0.458 | 1.23, ns |
| SA | 2.986 | 3.092 | 8.99, $p < 0.005$ |

*Note*: CPYDS-12: global indicator comprising bonding, resilience, social competence, emotional competence, cognitive competence, moral competence, self-efficacy, beliefs about the future, spirituality, clear and positive identity, prosocial involvement, and recognition for positive behavior. CPYDS-10: global indicator comprising resilience, social competence, emotional competence, cognitive competence, behavioral competence, moral competence, self-determination, self-efficacy, beliefs in the future, and clear and positive identity. KEY15: indicator based on 15 key items. KEY36: indicator based on 36 items. SEC: social-emotional competence indicator based on Factor 1 and Factor 2. DE: Delinquency Scale. SA: school adjustment behavior.

showed similar, but stronger, results. The present findings showed that the effect of the program was positive in the first 2 years of junior secondary years. In conjunction with the previous findings based on objective outcome evaluation, subjective outcome evaluation, qualitative evaluation via focus groups, qualitative evaluation via diaries, process evaluation, and interim evaluation, the existing evaluation findings from the Project P.A.T.H.S. basically suggest that the program is an effective one. In view of the paucity of outcome studies in Hong Kong, the present study contributes to evidence-based youth work in Hong Kong[14].

Although the present findings provide support for the Project P.A.T.H.S. using longitudinal findings, several limitations of the study should be noted. First, the effect size associated with the significant finding was on the low side. This observation may be due to the fact that the duration of the program was still short. Second, as only 2 years were involved in the program, only the short-term effect of the program was revealed. Obviously, it is important to evaluate the long-term effect of the program. Third, while analyses of covariance and linear mixed models are commonly used to analyze effectiveness of intervention programs, analyses based on growth curves should be used to examine the differences between experimental and control participants.

### ACKNOWLEDGMENTS

The preparation for this paper and the Project P.A.T.H.S. were financially supported by The Hong Kong Jockey Club Charities Trust.
